# Prenatal stress induces a depressive-like phenotype in adolescent rats: The key role of TGF-β1 pathway

**DOI:** 10.3389/fphar.2022.1075746

**Published:** 2022-12-02

**Authors:** Annamaria Fidilio, Margherita Grasso, Giuseppe Caruso, Nicolò Musso, Veronica Begni, Anna Privitera, Sebastiano Alfio Torrisi, Patrizia Campolongo, Stefania Schiavone, Fabio Tascedda, Gian Marco Leggio, Filippo Drago, Marco Andrea Riva, Filippo Caraci

**Affiliations:** ^1^ Unit of Neuropharmacology and Translational Neurosciences, Oasi Research Institute-IRCCS, Troina, Italy; ^2^ Department of Drug and Health Sciences, University of Catania, Catania, Italy; ^3^ Department of Biomedical and Biotechnological Sciences, University of Catania, Catania, Italy; ^4^ Bio-nanotech Research and Innovation Tower (BRIT), University of Catania, Catania, Italy; ^5^ Department of Pharmacological and Biomolecular Sciences, University of Milano, Milan, Italy; ^6^ Department of Physiology and Pharmacology “Vittorio Erspamer”, Sapienza University of Rome, Rome, Italy; ^7^ Neurobiology of Behavior Laboratory, Santa Lucia Foundation, Rome, Italy; ^8^ Department of Clinical and Experimental Medicine, University of Foggia, Foggia, Italy; ^9^ Department of Life Sciences, University of Modena and Reggio Emilia, Modena, Italy; ^10^ Biological Psychiatry Unit, IRCCS Istituto Centro San Giovanni di Dio Fatebenefratelli, Brescia, Italy

**Keywords:** depression, memory, TGF-β1, oxidative stress, prenatal stress

## Abstract

Stressful experiences early in life, especially in the prenatal period, can increase the risk to develop depression during adolescence. However, there may be important qualitative and quantitative differences in outcome of prenatal stress (PNS), where some individuals exposed to PNS are vulnerable and develop a depressive-like phenotype, while others appear to be resilient. PNS exposure, a well-established rat model of early life stress, is known to increase vulnerability to depression and a recent study demonstrated a strong interaction between transforming growth factor-β1 (TGF-β1) gene and PNS in the pathogenesis of depression. Moreover, it is well-known that the exposure to early life stress experiences induces brain oxidative damage by increasing nitric oxide levels and decreasing antioxidant factors. In the present work, we examined the role of TGF-β1 pathway in an animal model of adolescent depression induced by PNS obtained by exposing pregnant females to a stressful condition during the last week of gestation. We performed behavioral tests to identify vulnerable or resilient subjects in the obtained litters (postnatal day, PND > 35) and we carried out molecular analyses on hippocampus, a brain area with a key role in the pathogenesis of depression. We found that female, but not male, PNS adolescent rats exhibited a depressive-like behavior in forced swim test (FST), whereas both male and female PNS rats showed a deficit of recognition memory as assessed by novel object recognition test (NOR). Interestingly, we found an increased expression of type 2 TGF-β1 receptor (TGFβ-R2) in the hippocampus of both male and female resilient PNS rats, with higher plasma TGF-β1 levels in male, but not in female, PNS rats. Furthermore, PNS induced the activation of oxidative stress pathways by increasing inducible nitric oxide synthase (iNOS), NADPH oxidase 1 (NOX1) and NOX2 levels in the hippocampus of both male and female PNS adolescent rats. Our data suggest that high levels of TGF-β1 and its receptor TGFβ-R2 can significantly increase the resiliency of adolescent rats to PNS, suggesting that TGF-β1 pathway might represent a novel pharmacological target to prevent adolescent depression in rats.

## 1 Introduction

Major depressive disorder (MDD) is one of the most common mood disorder characterized by affective and cognitive symptoms affecting the life’s quality of patients (Bhatt et al. (2020) with a prevalence of 5.7% in children and 11.3% in adolescents (Bowman and Daws 2019). Recent studies also show that the rates of depression increase after puberty in girls more than in boys (Silberg et al., 1999). Although depression in adolescents could be considered as a familial disorder resulting in approximately 37% of heritability, where genetic factors have a strong role in this transmission, environmental factors play a key role in the development of adolescent depression (Zalsman et al., 2006; Thapar et al., 2012). Several epidemiological studies demonstrate that the exposure to adverse experiences during pregnancy and stressful early life conditions during childhood can increase the vulnerability to depression (Laplante et al., 2008; Vedhara et al., 2012). Nevertheless, not all individuals exposed to prenatal or postnatal stress develop depressive-like phenotypes, but they can exhibit a notable degree of resilience (Russo et al., 2012). Different studies have been conducted in the last ten years to identify the genetic and molecular mechanisms underlying resilience, but discrepancies exist between human and animal studies in this field, and animal models remain an essential tool to investigate which factors may be involved in the stress resilience mechanisms (Russo et al., 2012). According to this scenario, animal models of prenatal stress (PNS) are used to reproduce the effects of early adverse life events in humans during early life and represent a useful tool to examine the long-term effects of stressful events during pregnancy and to identify the molecular mechanisms underlying vulnerability/resilience to depression in adolescence (Maccari et al., 2014).

PNS exposure is known to increase vulnerability to depression in adult rats (Cattaneo et al., 2020) and different studies have demonstrated that adult animals exposed to PNS procedure show depressive-like or anxious-like phenotypes paralleled by hypothalamic-pituitary-adrenal (HPA) axis dysfunction and an impairment of neuronal plasticity mechanisms (Fumagalli et al., 2004; Maccari and Morley-Fletcher 2007; Luoni et al., 2015; Caruso et al., 2019c). Oxidative stress plays a key role in the pathogenesis of depression by the reduction of antioxidant enzymes and by increasing reactive oxygen species (ROS) production (Bajpai et al., 2014; Caruso et al., 2019a; Caruso et al., 2019c; Bhatt et al., 2020). Starting from the evidence obtained in pediatric and adolescent patients with depressive disorders of increased blood levels of oxidative stress markers combined with a reduction in antioxidant factors, such as superoxide dismutase and glutathione peroxidase (Katrenčíková et al., 2021), it can be hypothesized an activation of oxidative and nitrosative stress pathways in animal models of adolescent depression. It has been suggested a strong neurobiological link between early life adverse experiences (e.g., maternal separation and social isolation) and redox state dysfunctions in PNS-induced depression (Schiavone et al., 2013), and also that stress-related conditions are followed by changes in the pro-oxidant/antioxidant ratio in various tissues (Liu et al., 2015; Moniczewski et al., 2015).

Depressed children and adolescents have shown HPA axis dysregulation and higher basal cortisol levels compared to healthy controls (Silberg et al., 1999). This enhanced cortisol secretion is associated with: i) increased oxidative tissue damage in adolescent children with depression (Oravcova et al., 2022); ii) a deficit of serotoninergic system (Hughes et al., 1996) combined with a lower expression/function of the serotonin transporter than in adults and a reduced clinical response to selective serotonin reuptake inhibitors (SSRIs) (Bowman And Daws 2019).

It is also well-known that chronic stress can act as a risk factor for the development of MDD through the impairment of neurotrophins signaling such as brain-derived neurotrophic factor (BDNF) and transforming growth factor-β1 (TGF-β1) (Pittenger and Duman 2008; Guerrera et al., 2020). Adult rats subjected to PNS *in utero* show a reduction of BDNF levels in the prefrontal cortex (Luoni et al., 2014).

An impairment of TGF-β1 was observed in animal models of depression with cognitive dysfunction (Yu et al., 2011), whereas a reduction of plasma TGF-β1 levels was detected in depressed patients and correlated with depression symptoms severity and treatment resistance (Myint et al., 2005; Rush et al., 2016; Caraci et al., 2018a). We have recently demonstrated a deficit of hippocampal TGF-β1 levels paralleled by a depressive-like phenotype and cognitive impairment in an animal model of amyloid-related depression, whereas no studies have been conducted yet in animal models of adolescent depression (Applebaum and Wilson 1988). Interestingly, recent studies in humans have provided a strong evidence for a key role of TGF-β1 signaling in depression (Cattaneo et al., 2018; Qiu et al., 2021). A first study has examined the transcriptome and miRNome profiles from the hippocampus of adult rats exposed to PNS with transcriptome data obtained from blood mRNA of adult humans exposed to early life trauma, demonstrating a strong interaction between TGF-β1 gene and PNS in the regulation of mechanisms relevant to stress and depression (Cattaneo et al., 2018). A second study analyzed the TGF-β1 SMAD-dependent pathway in the neurodevelopment of children exposed to maternal depression *in utero* showing that children with a lower gene expression score of TGF-β type I transmembrane receptor (TGF-βRI) exhibited larger amygdala volumes in relation to greater prenatal maternal depressive symptoms (Qiu et al., 2021). An open question remains to understand whether a deficit of TGF-β1 signaling might increase the vulnerability to depression in children and adolescence and, most importantly, whether TGF-β1 signaling might be studied as a new pharmacological target for the prevention or treatment of maternal depression during pregnancy, with a positive effect in the offspring in the context of brain development (Qiu et al., 2021). TGF-β1 plays also a key role in cognitive dysfunction in depression (Caraci et al., 2018b), but it is presently unknown the role of TGF-β1 pathway in animal models of adolescent depression induced by PNS.

Starting from the above evidence, we used an animal model of PNS in pregnancy, obtained by exposing pregnant females to a stressful condition during the last week of gestation, in order to: i) assess whether it can induce the development of a depressive-like phenotype in offspring during adolescence; ii) to identify the molecular mechanisms mediating the vulnerability or resilience to PNS. In particular, we hypothesized that an impairment of the TGF-β1 pathway and oxidative stress increase the vulnerability to adolescent depression induced by PNS. In the present work, we show, for the first time, that an increased response of the TGF-β1 pathway due to PNS can increase the resiliency to adolescent depression.

## 2 Materials and methods

### 2.1 Animals

A total of 26 adult male (weight 400 g) and female (weight 250 g) Sprague-Dawley rats were obtained from Envigo RMS s. r.l. laboratories (San Pietro al Natisone, Italy) and group-housed for 8 days after arrival to eliminate stress from shipping. Then, nulliparous female rats were separately housed in the presence of sexually experienced male rats and a vaginal smear was performed daily, by introducing a small amount of saline into the vagina using a pipette and placing drops of the cell suspension onto a slide and by labeling with methylene blue, to check by light microscopy the presence of sperm and to establish the first day of pregnancy. Pregnant female rats, randomly assigned to control and PNS groups, were individually housed with access to food and water *ad libitum* and maintained in an air-conditioned (23°C ± 1°C) room with constant humidity (60% ± 10%) and a 12 h light/12 h dark cycle.

### 2.2 PNS model

**FIGURE 1:**
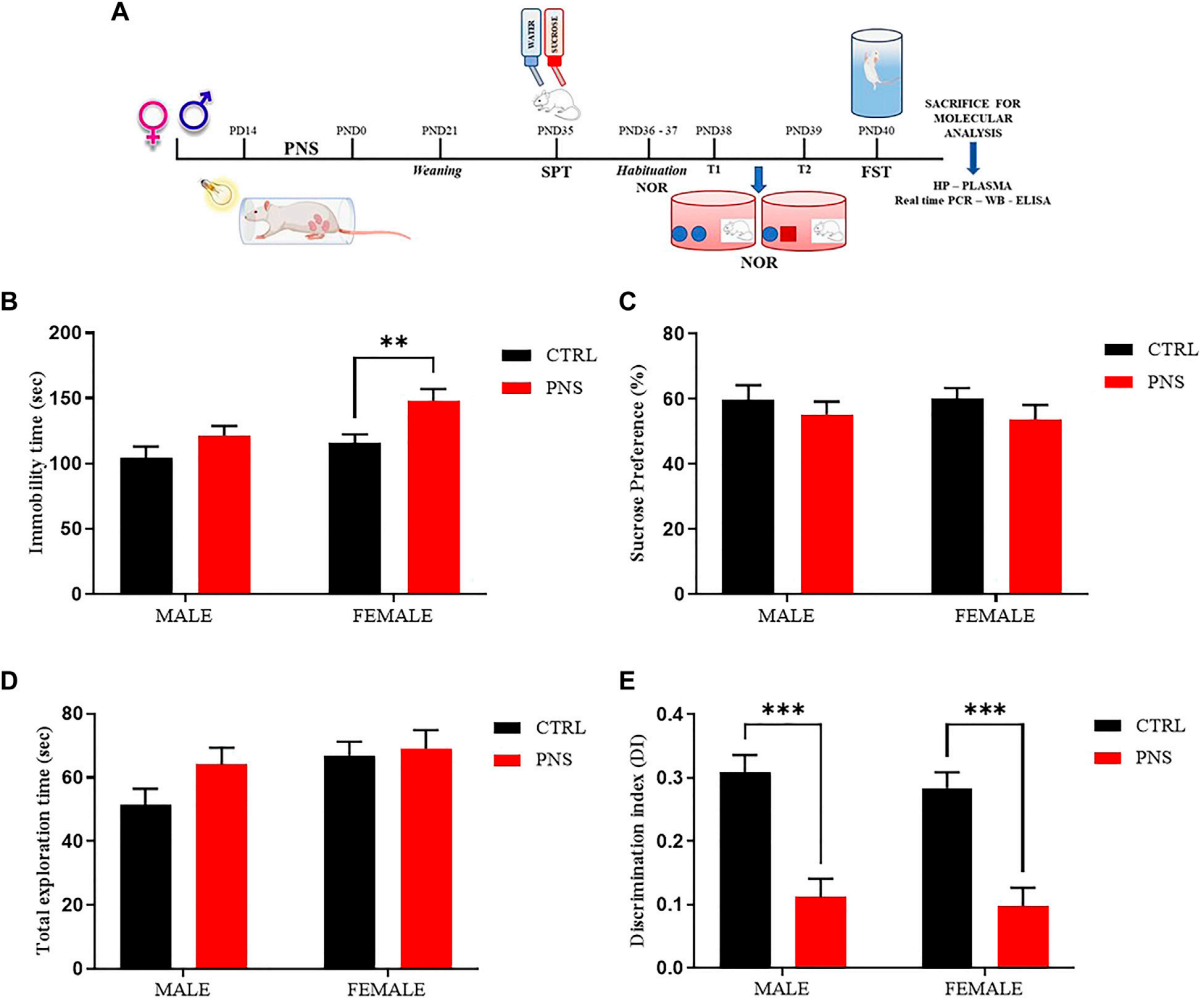
PNS induces depressive-like behavior and recognition memory deficits in adolescent rats. **(A)** Schematic representation of the experimental design. CTRL = Control, PNS = Prenatal Stress, PD = Pregnancy Day, PND = Post Natal Day, SPT = Sucrose Preference Test, NOR = Novel Object Recognition, FST = Forced Swim Test, Real time-PCR = Real time-Polymerase Chain Reaction, WB = Western Blot, ELISA = Enzyme-linked immunosorbent assay, and HP = Hippocampus. **(B)** Depressive-like behavior assessed in the FST by measuring immobility time (sec) in PNS and CTRL rats. **p < 0.01 vs. CTRL female. **(C)** Sucrose preference (%) of CTRL and PNS males and female rats. **(D)** Total Exploration Time and **(E)** Discrimination Index (D.I) were used to evaluate recognition memory in rats. ***p < 0.001 vs. CTRL groups. All data are shown as mean ± SEM of CTRL male *n* = 29; CTRL female *n* = 43; PNS male *n* = 37; PNS female *n* = 25.

The experimental design carried out in this study is reported in Figure 1A. We used a PNS model obtained by exposing pregnant dams to a stressful condition during the last week of gestation as previously described (Luoni et al., 2014; Marchisella et al., 2021). Briefly, PNS pregnant female rats were placed into plexiglass transparent cylinders (9 cm diameter, 9 cm height, and 20 cm length) and exposed to bright light (1,500 lux) for 45 min, three times a day (09:00 a.m.–12:00 a.m.–17:00 p.m. ± 2 h), at different periods of the day in order to reduce possible habituation to repeated stress. Control pregnant female rats were left undisturbed in home cages. Male and female offspring (CTRL male *n* = 29; CTRL female *n* = 43; PNS male *n* = 37; PNS female *n* = 25) were weaned 21 days after birth, randomly housed in the same sex groups of 2 or 3 per cage and maintained under control conditions. Then animals were handled daily before behavioral test sessions.

### 2.3 Behavioral assessment

We performed specific behavioral tests to assess anhedonia-like behavior, cognitive functions, and depressive-like behavior in male and female offspring during adolescence (PND 35-40) from stressed and control dams in order to identify vulnerable (V) and resilient (R) litters. At the end of behavioral characterization, animals were sacrificed at PND 47 for molecular investigations in hippocampus and plasma in order to identify possible mechanisms underlying the vulnerability and/or the resilience to PNS (Figure 1A).

#### 2.3.1 Sucrose preference test

In order to assess anhedonia-like behavior we performed the Sucrose Preference Test (SPT) as previously described by Marchisella et al. (2021). Briefly, the night before the test (PND 34), adolescent PNS and control rats, habituated to having two water bottles during normal maintenance conditions, were singly housed and water deprived for 12 h. The next day (PND 35), all animals were given 3 h access to one bottle of 1% (wt/vol) sucrose solution and one bottle of regular water. Each bottle was weighed before and after the test. At the end of the test, all animals were group-housed again with food and water *ad libitum*. Anhedonia-like behavior, as a reduction in sucrose preference ratio relative to control group, was evaluated using the following formula: sucrose preference = (sucrose intake/total intake) *100, with the total intake as the sum of sucrose and water intake (in grams).

#### 2.3.2 Novel object recognition test

The Novel Object Recognition (NOR) test, a paradigm for the investigation of recognition memory in preclinical field, was performed as previously described by Mhillaj et al. (2018). All animals (PND 36-39) were transferred to the test room 30 min prior to the experiment’s start to allow the acclimation. During the NOR test, rats received two 5 min-habituation sessions to explore the circular arena (75 cm diameter, 33 cm height). In the first trial (T1) day test, rats were submitted to a 10-min exposure to two identical (familiar) objects placed in the central part of the arena equally distant from the perimeter, and then animals returned to their home cage. The next day (T2), rats were exposed to one familiar object (F) and to one novel object (N) for 10 min. After each trial, arena and objects were cleaned with a 50% ethanol solution and dried. We waited 10 min to allow the evaporation of ethanol solution, avoiding the presence of possible olfactory cues. The number of exploration seconds of each object was recorded. Exploration was defined as sniffing and touching the object with the nose and quantified as *total exploration time = TN (sec) + TF (sec)* and *discrimination index (D.I.) = (TN – TF)/TN + TF.* A reduction in discrimination index ratio relative to control group indicates a recognition memory impairment.

#### 2.3.3 Forced swim test (FST)

In order to evaluate the possible depressive-like behavior caused by PNS procedure in offspring, we performed the Forced Swim Test (FST) in adolescent animals (PND 40) according to the original protocol employed by Porsolt et al. (1978) with slight modifications following the published protocol by Monteggia et al. (2007). All animals were transferred to the test room 30 min prior to the experiment’s start to allow the acclimation. PNS and control rats were placed in a plexiglass transparent cylinder tank containing 2/3 of temperature controlled water (24°C ± 1°C) for a total time of 6 min. Water was changed after each test and tested animals were dried with a clean paper towel and placed underneath a heat source (37°C) in order to restore their body temperature. After an initial habituation period of 2 min, the mobility, defined as any movements other than those necessary to balance the body and keep the head above the water (Cryan et al., 2002), and immobility time was recorded during the last 4 min of the test. An increase in the immobility time relative to control group, evaluated with the following formula: *Immobility (sec) = 240 sec– mobility time (sec)*, indicates a depressive-like behaviour.

### 2.4 Behavioral z-scoring

To identify vulnerable and resilient rats obtained by PNS procedure we applied the z-normalization (*Total z-score*), a simple mathematical and integrative tool widely used in clinical and preclinical studies (Guilloux et al., 2011) to measure cognitive and emotional dimensions, generated from mean of every z-score value obtained from different tests. This allows all parameters to be of the same magnitude, so that a possible outlier in the dataset has been transformed to prevent it from being a massive outlier, and thus directly comparable. The benefit of performing this type of normalization is to reduce the behavioral noise related to the use of multiple tests (Torrisi et al., 2021).

*Z-score* indicates how many standard deviations (σ) an individual observation (X) is above or below the mean of a control group (μ): *Z-score = (X–μ)/σ*.

After calculating the z-score for each behavioral test, we measured the z-normalization *Total z-score* for each animal by using the following formula: *Total z-score = (z-score SPT + z-score NOR + z-score FST)/(n) behavioral tests*, with the aim to classify vulnerable and resilient animals for both emotional and cognitive dimensions. PNS rats were separated into resilient and vulnerable on the basis of the results obtained on three different behavioral tests, which assess depressive- (FST) and anhedonia- (SPT) like behavior and cognitive memory deficit (NOR). A rat was considered to have a PNS- induced depressive-like phenotype when the mean performance resulting from all three different tests differed by ½ standard deviation (SD) from the mean level of the performance obtained by the control group.

### 2.5 Gene expression analysis by quantitative real-time PCR

The extraction of total RNA from each hippocampal sample was performed as previously described (Marchisella et al., 2021), with slight modifications. Briefly, tissue samples were homogenized in a homogenization buffer, containing sucrose, phenylmethylsulfonyl fluoride, HEPES, MgCl_2_, NaHCO, cOmplete (protease inhibitor cocktail tablets, EDTA-free, Sigma-Aldrich, 11836170001), phosphatase and RNase inhibitors (Sigma-Aldrich, St. Louis, MO, United States), and sterile H_2_O, by using ULTRA-TURRAX homogenizer (IKA^®^-Werke GmbH & Co. KG, Darmstadt, Germany). Once completely homogenized, a half of each sample was used for RNA extraction, while the other half was used for protein extraction.

In order to extract the RNA from each hippocampal sample, homogenized solutions were sonicated, added with PureZOL (Bio-Rad Laboratories, Inc., Milan, Italy), and chloroform, and centrifuged (12,000 rpm at 4°C for 15 min). The aqueous phase was transferred into a new vial and incubated with isopropanol overnight at −20°C. The day after, each solution was centrifuged (12,000 rpm at 4°C for 30 min), the supernatant was discarded, an appropriate quantity of 75% ethanol solution was added followed by centrifugation (12,000 rpm at 4°C for 15 min), the supernatant was discarded, and the pellet was left to dry at room temperature for 5 min. The pellet was then resuspended in an adequate volume of RNase-Free Water (Qiagen, Hilden, Germany).

Total RNA concentration was assessed by using NanoDrop^®^ ND-1000 (Thermo Fisher Scientific, Waltham, MA, United States), while RNA quality was determined using Qubit^®^ 3.0 Fluorometer (Thermo Fisher Scientific) (Fresta et al., 2020a). The reverse transcription of 1 μg of RNA (for each sample) was accomplished by using QuantiTect Reverse Transcription Kit (Qiagen) according to manufacturer instructions, while the quantification of each cDNA sample loaded in a 384-well plate was obtained by employing a LightCycler^®^ 480 System (Roche Molecular Systems, Inc., Pleasanton, CA, United States). The primer sequences (QuantiTect Primer Assays, Qiagen) used for gene expression analysis are shown in Table 1, with the exception of those for NOX1 (forward: 5′-CTT CCT CAC TGG CTG GGA TA-3′; reverse: 5′-CGA CAG CAT TTG CGC AGG CT-3′) (Uchizono et al., 2006) that were purchased by Life Technologies Italia (Monza, Italy). The protocol used to achieve sample amplification, fluorescence data collection, and sample quantification is the same as previously described (Caruso et al., 2019b; Fresta et al., 2020b). Glyceraldehyde-3-phosphate dehydrogenase (GAPDH) and *β*-actin were selected as the combination of reference genes.

**TABLE 1:** The list of primers used for quantitative real-time PCR (qRT-PCR).

Official name^#^	Official symbol	Alternative titles/symbols	Detected transcript	Amplicon length	Cat. No.^§^
nitric oxide synthase 2, inducible	Nos2	iNos; Nos2a	NM_012611	118 bp	QT00178325
cytochrome b-245, beta polypeptide	Cybb	Nox2; Gp91-phox	NM_023965	64 bp	QT00195300
transforming growth factor, beta 1	Tgfb1	Tgfb; Tgfb-1; TGFbeta1; TGF-beta1	NM_011577	145 bp	QT00145250
transforming growth factor, beta receptor 2	Tgfbr2	Tgfbr2T; TGF-beta 2	NM_031132 XM_008766690	99 bp	QT00182315
glyceraldehyde-3-phosphate dehydrogenase	Gapdh	Gapd; BARS-38	NM_017008	149 bp	QT00199633
actin, beta	Actb	Actx	NM_031144	145 bp	QT00193473

^#^ https://www.ncbi.nlm.nih.gov/gene/, ^§^ https://www.qiagen.com/it/shop/pcr/real-time-pcr-enzymes-and-kits/two-step-qrt-pcr/quantitect-primer-assays/.

### 2.6 Western blot analysis

After initial homogenization using a homogenization buffer containing protease (cOmplete, Sigma-Aldrich, 11836170001) and phosphatase inhibitors cocktail (Sigma-Aldrich, P0044), part of each hippocampus sample isolated from PNS and control rats was used for Western Blot analysis as previously described (Fidilio et al., 2021). Briefly, samples were sonicated (10 sec, 3 cycles), centrifuged at 14,000 *g* at 4°C for 15 min and then the supernatants were collected. Protein concentrations were quantified by using a Pierce™ BCA protein assay kit with bovine serum albumin as a standard (Thermo Fisher Scientific, 23,227), according to the manufacturer’s specifications; subsequently, 20 μg of total proteins were denatured at 95 °C for 10 min, separated by NuPage™ 4%–12% bis–tris gel electrophoresis (Thermo Fisher Scientific, NP0322BOX) and transferred to nitrocellulose membranes. After a blocking step, the membranes were incubated overnight at 4°C with the following primary antibodies: rabbit anti-TGF-β1 (1:500, Abcam, Cambridge, United Kingdom; ab92486), rabbit anti-TGFβ-R2 (1:500, Cell signaling Technology Inc., Danvers, MA, United States; 79,424), rabbit anti-NOX1 (1:1,000, Abcam; ab131088), rabbit anti-NOX2 (1:4,000, Abcam; ab80508), rabbit anti-iNOS (1:500, Abcam; ab136918), and mouse anti-actin (1:1,000, Sigma-Aldrich, A4700) used as housekeeping. Secondary goat anti-rabbit labeled with IRDye 800CW (Li-COR Biosciences; 1:15,000) and goat anti-mouse labeled with IRDye 680LT (Li-COR Biosciences; 1:15,000) were used at room temperature for 60 min in the dark. Hybridization signals were detected using an Odyssey^®^ Infrared Imaging System (LI-COR Biosciences, Lincoln, NE, United States), and the densitometric analysis was performed by Image J software.

### 2.7 TGF-β1 measurement in plasma samples

In order to measure TGF-β1 levels in plasma samples obtained from vulnerable and resilient rats (both males and females) as well as in control rats, we carried out the enzyme-linked immunosorbent assay (ELISA) in accordance to manufacturer’s instructions (Bio-techne, R&D system, DB100C). After the activation procedure from latent TGF-β1 to the immunoreactive form, plasma samples were assayed at a 1:10 dilution. TGF-β1 standard curve and activated samples were assayed in duplicate. The sensitivity of the ELISA kit, as reported in the data sheet, is 15.4 pg/ml. The optical density of each well was determined using a microplate reader Synergy HT (Agilent BioTek, Santa Clara, CA, United States) set to 450 nm, 540 nm, and 570 nm as suggested by the producer. Data were analyzed subtracting readings at 540 nm from the readings at 450 nm in order to correct the optical imperfections in the plate.

### 2.8 Statistical analysis

All statistical analyses were carried out by using the software GraphPad Prism^®^, version 9.0 (GraphPad, La Jolla, CA, United States). Results obtained by behavioral assessment were analyzed using two-way Analysis Of Variance (ANOVA), followed by Bonferroni’s *post hoc* multiple comparisons, while data from molecular investigations were analyzed by using one-way ANOVA followed by Bonferroni’s *post-hoc* test. Data were expressed as mean ± Standard Error of Mean (SEM) and only *p*-values < 0.05 were considered statistically significant. GraphPad Prism^®^, version 9.0 was also used to identify possible outliers in all experimental groups. The Spearman rank test was used for bivariate correlation analysis between peripheral and central TGF-β1 levels in both male and female PNS rats.

### 2.9 Study approval

The study was authorized by the Institutional Animal Care and Use Committee (IACUC) of the University of Catania and by the Italian Ministry of Health (DDL 26/2014 and previous legislation; OPBA Project #337/2020; Authorization n. 300/2020-PR). Animal care followed Italian (D.M. 116192) and EEC (O.J. of E.C.L 358/1 12/18/1986) regulations on protection of animals used for experimental and scientific purposes.

## 3 Results

### 3.1 PNS exposure induces a depressive-like behavior during adolescence only in females, but causes a deficit in recognition memory in both sexes

To assess if PNS procedure contributes to emotional impairment, FST and SPT were adopted to evaluate a possible depressive- and anhedonia-like behavior, respectively. Figure 1B shows an increase of immobility time in female rats exposed to PNS if compared to the control group (*p* < 0.01); whereas no significant changes were observed in immobility time in male PNS and CTRL rats. We also explored the anhedonic-like behaviour by employing SPT and we found only a small decrease in sucrose preference, but not statistically relevant, in both male and female PNS rats compared to the control group (Figure 1C).

In order to extend our investigation to the effect of PNS on cognitive dimension, we tested adolescent rats using NOR test, a task based on the natural tendency of rodents to explore unfamiliar objects, which depends upon integrity of the perirhinal cortex, the hippocampus, and the medial temporal lobe (Broadbent et al., 2010). Figure 1E reveals a strong significant reduction of discrimination index values in both male and female PNS rats compared to the control group (*p* < 0.001), suggesting that PNS animals were not able to keep the information acquired during the T1 training trial and they did not discriminate between the familiar and the novel object compared to control rats. Results were not affected by differences in total exploration time between all experimental animal groups (Figure 1D).

These data show for the first time that PNS exposure induces an impairment of object recognition memory both in male and female adolescent rats and a depressive-like behavior only in adolescent female rats, suggesting that PNS is able to cause a strong deficit in a cognitive dimension, also relevant in humans, and a gender difference in emotional response.

### 3.2 Characterization of vulnerable and resilient rats exposed to PNS using total z-score

**FIGURE 2:**
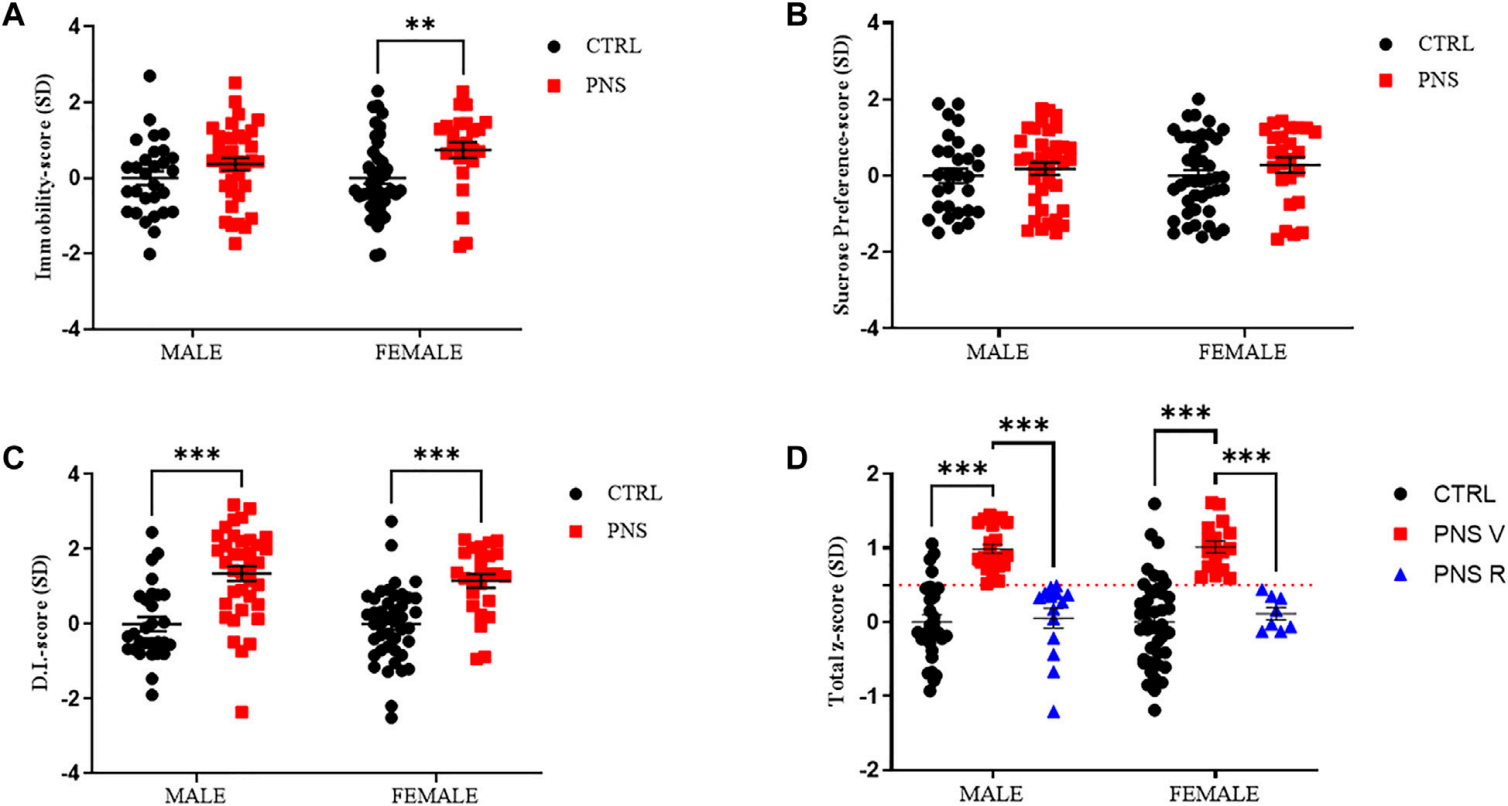
Identification of vulnerable and resilient rats by applying z-score method. **(A)** z-score values in CTRL and PNS male and female adolescent rats in FST. **p < 0.01 vs. CTRL female. **(B)** z-score values in CTRL and PNS male and female adolescent rats in SPT. **(C)** z-score values in CTRL and PNS male and female adolescent rats in NOR test. ***p < 0.001 vs. CTRL groups. **(D)** Total z-score = (Z-score FST + Z-score SPT + Z-score NOR)/3). Male PNS V = 62%, ***p < 0.001 vs. CTRL and PNS R; female PNS V = 68%, ***p < 0.001 vs. CTRL and PNS R.

To identify vulnerable and resilient rats exposed to PNS procedure, we analyzed both emotional and cognitive behavioral data using the z-normalization. Z-score values of single behavioral tests indicate that PNS rats showed an increase of immobility time (Figure 2A, male PNS V = 33%; female PNS V = 44%, *p* < 0.01), a significant reduction in discrimination index (Figure 2C, male PNS V = 65%, *p* < 0.001; female PNS V = 68%, *p* < 0.001), without significant changes in sucrose preference intake (Figure 2B), compared to the control group. Conversely, resilient rats exhibited a similar behavioral response to the control animals. After calculating the z-score of FST, SPT, and NOR (Figures 2A,B,C), we applied z-normalization (Total z-score) (Figure 2D) for all behavioral data using the formula: *Total z-score = (Z-score FST + Z-score SPT + Z-score NOR)/3* (see above paragraph 2.4). Z-normalization data reveal a slightly higher percentage of vulnerability in female rats than males exposed to PNS procedure (male PNS V = 62%, *p* < 0.001 vs. CTRL and PNS R; female PNS V = 68%, *p* < 0.001 vs. CTRL and PNS R). By applying the z-normalization, we obtained *n* = 23 and *n* = 14 PNS males V and R, respectively, while *n* = 17 and *n* = 8 were obtained in the case of PNS females V and R, respectively (Figure 2D).

### 3.3 Molecular mechanisms underlying vulnerability to depression and memory deficits in PNS rats: A key role of TGF-β1

**FIGURE 3:**
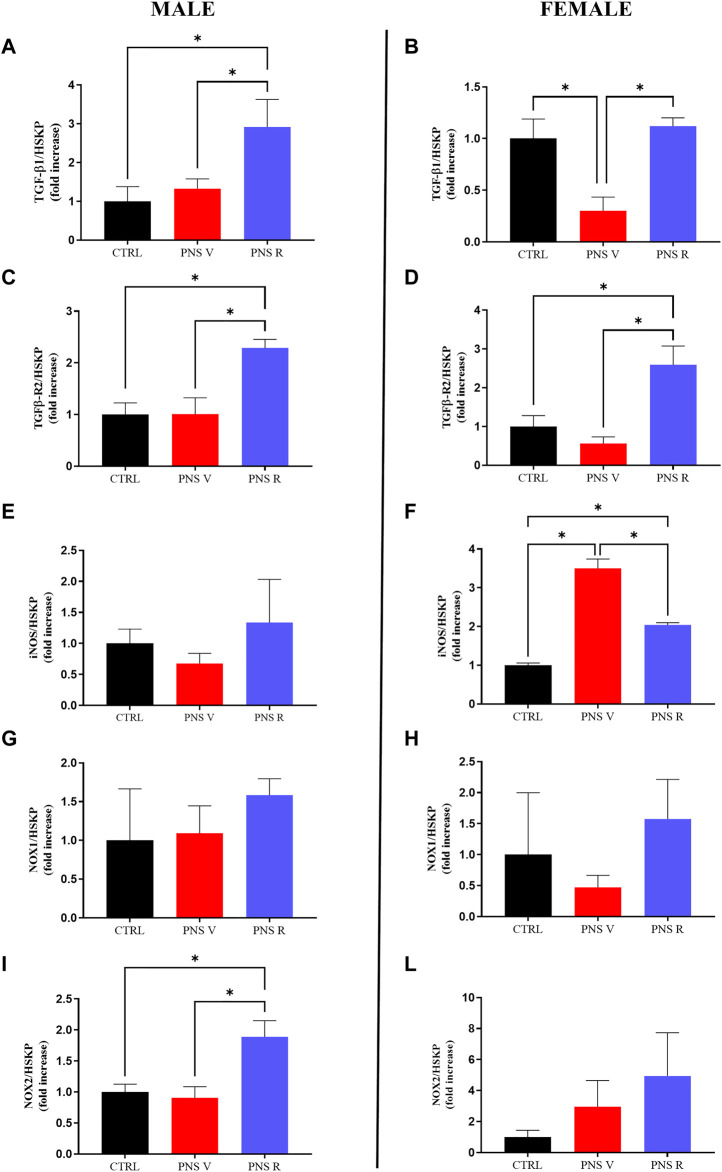
Effects of PNS on gene expression in the hippocampus: impact on TGF-β1 pathway and oxidative stress machinery. Effects induced by PNS exposure on **(A,B)** TGF-β1, **(C,D)** TGFβ-R2, **(E,F)** iNOS, **(G,H)** NOX1, and **(I,J)** NOX2 mRNAs expression measured by qRT-PCR. The abundance of each mRNA of interest was expressed relative to the abundance of GAPDH/β-actin (HSKP) mRNA, as endogenous controls. As a negative control, a reaction in absence of cDNA (no template control, NTC) was performed. qRT-PCR amplifications were performed at least in triplicate. Data are shown as mean ± S.E.M. *p < 0.05.

Starting from the evidence that chronic stress, by impairing TGF-β1 pathway and promoting oxidative stress, can represent a risk factor for the development of depression (Caraci et al., 2018b; Cattaneo et al., 2018; Caruso et al., 2021b), we first examined the role of TGF-β1 signaling in mediating the PNS response by evaluating the mRNA levels of this neurotrophic factor and its receptor TGFβ-R2 in the hippocampus of animals exposed to PNS procedure (Figures 3A–D) in parallel to the analysis of the pro-oxidant enzymes inducible nitric oxide synthase (iNOS), responsible for nitric oxide production (Metto et al., 2013), and NADPH oxidase 1 (NOX1) and 2 (NOX2), responsible for superoxide production (de Campos et al., 2015; Ogboo et al., 2022) (Figures 3E,I).

Interestingly, we found that PNS exposure reduced the expression of TGF-β1 gene only in the hippocampus of vulnerable female (PNS V) (*p* < 0.05 vs. CTRL and PNS R), but not in PNS V male, whereas TGF-β1 mRNA levels were comparable to that of controls in resilient female (PNS R) and significantly increased (*p* < 0.05) compared to controls in resilient male (PNS R) (Figures 3A,B). Recent studies in depressed patients suggest that TGF-β1 receptor genes play a central role in the pathogenesis of child and adolescent depression (Qiu et al., 2021). Interestingly, along this line, we observed, in our model of adolescent depression, significantly increased TGFβ-R2 mRNA levels only in resilient male and female when compared both to controls and vulnerable rats (Figures 3C,D; *p* < 0.05), suggesting a key role of TGF-β1 pathway in the pathophysiology of adolescent depression.

In our *in vivo* model we found that PNS induced a significant increase of iNOS mRNA levels in PNS V females compared to controls and PNS R (Figure 3F; *p* < 0.05). With regard to NOX2 mRNA levels, it was observed that the expression of this gene was significantly increased in PNS R males compared to both controls and PNS V (Figure 3I; *p* < 0.05). A different effect was observed when considering the mRNA expression levels of NOX2 in female rats, where a trend of increase was observed in PNS V and PNS R compared to controls (Figure 3J). In the case of NOX1, a trend of increase was observed for PNS R males only compared to both PNS V and controls (Figure 3G), while no significant differences in the expression of this gene were detected among the different experimental groups in the case of female rats (Figure 3H).

**FIGURE 4:**
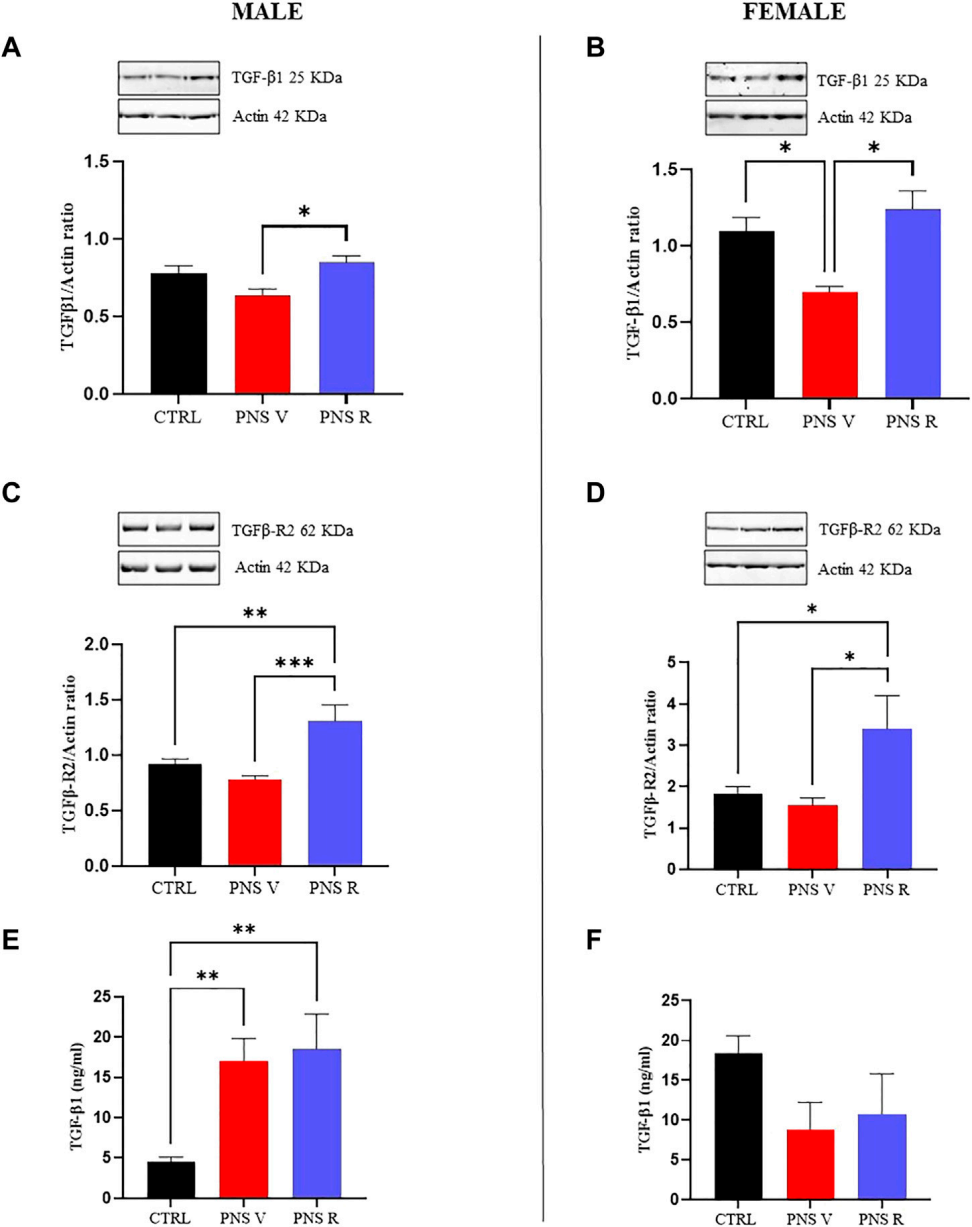
Role of TGF-β1 pathway in the mechanisms of vulnerability and resilience to PNS exposure. Effects induced by PNS exposure on TGF-β1 levels and of its receptor TGFβ-R2 in total protein extracts from hippocampus of CTRL and PNS rats evaluated by Western Blot analysis. **(A)** Representative immunoblot and histogram of TGF-β1 (44 kDa) in CTRL and PNS male adolescent rats (*p < 0.05 vs. PNS V). **(B)** Representative immunoblot and histogram of TGF-β1 (44 kDa) in CTRL and PNS female adolescent rats (*p < 0.05 vs. CTRL; *p < 0.05 vs. PNS V). **(C)** Representative immunoblot and histogram of TGFβ-R2 (65 kDa) in CTRL and PNS male adolescent rats (**p < 0.01 vs. CTRL; ***p < 0.001 vs. PNS V). **(D)** Representative immunoblot and histogram of TGFβ-R2 (65 kDa) in CTRL and PNS female adolescent rats (*p < 0.05 vs. CTRL; *p < 0.05 vs. PNS V). TGF-β1 and TGFβ-R2 densitometric values were normalized against actin used as internal control. Plasmatic levels of TGF-β1 (ng/ml) in male **(E)** (**p < 0.01 vs. CTRL) and female **(F)**. All data are shown as mean ± SEM of CTRL male *n* = 10; PNS V male *n* = 9; PNS R male *n* = 5; CTRL female *n* = 10; PNS V female *n* = 5; PNS R female *n* = 4.

To better understand the alterations of the TGF-β1 pathway in our model of adolescent depression, we carried out western blot analysis in hippocampus of control and PNS rats, also starting from the well-known evidence that TGF-β1 final activity is regulated not only at a transcriptional level, but also at a post-transcriptional level and primarily through the conversion of latent TGF-β1 to active TGF-β1 by a variety of proteases (Annes et al., 2003). Figure 4 showed a significant increase of TGF-β1 expression in both male and female PNS resilient rats (PNS R) compared to PNS V rats (Figures 4A,B; *p* < 0.05). Furthermore, we confirmed at a protein level a statistically significant deficit of TGF-β1 in female PNS V compared to both control and PNS R rats (Figure 4B; *p* < 0.05). When examining the protein levels of TGF-β1 receptor (TGFβ-R2), it was observed a significant increase in the expression of TGFβ-R2 in both male and female PNS R rats (Figures 4C,D) compared to PNS V (*p* < 0.001 for males; *p* < 0.05 for females) and control (*p* < 0.01 for males; *p* < 0.05 for females) groups. Finally, we examined the levels of active TGF-β1 in the plasma from control and PNS rats and we found an increase of TGF-β1 plasmatic levels only in male PNS V and PNS R rats compared to control group (Figure 4E; *p* < 0.01). On the contrary, in PNS female rats we observed a reduction, although not statistically significant, of this neurotrophic factor, but not an increase in females PNS R compared to control group (Figure 4F). No correlation was found between peripheral and central TGF-β1 levels in both male and female PNS rats.

**FIGURE 5:**
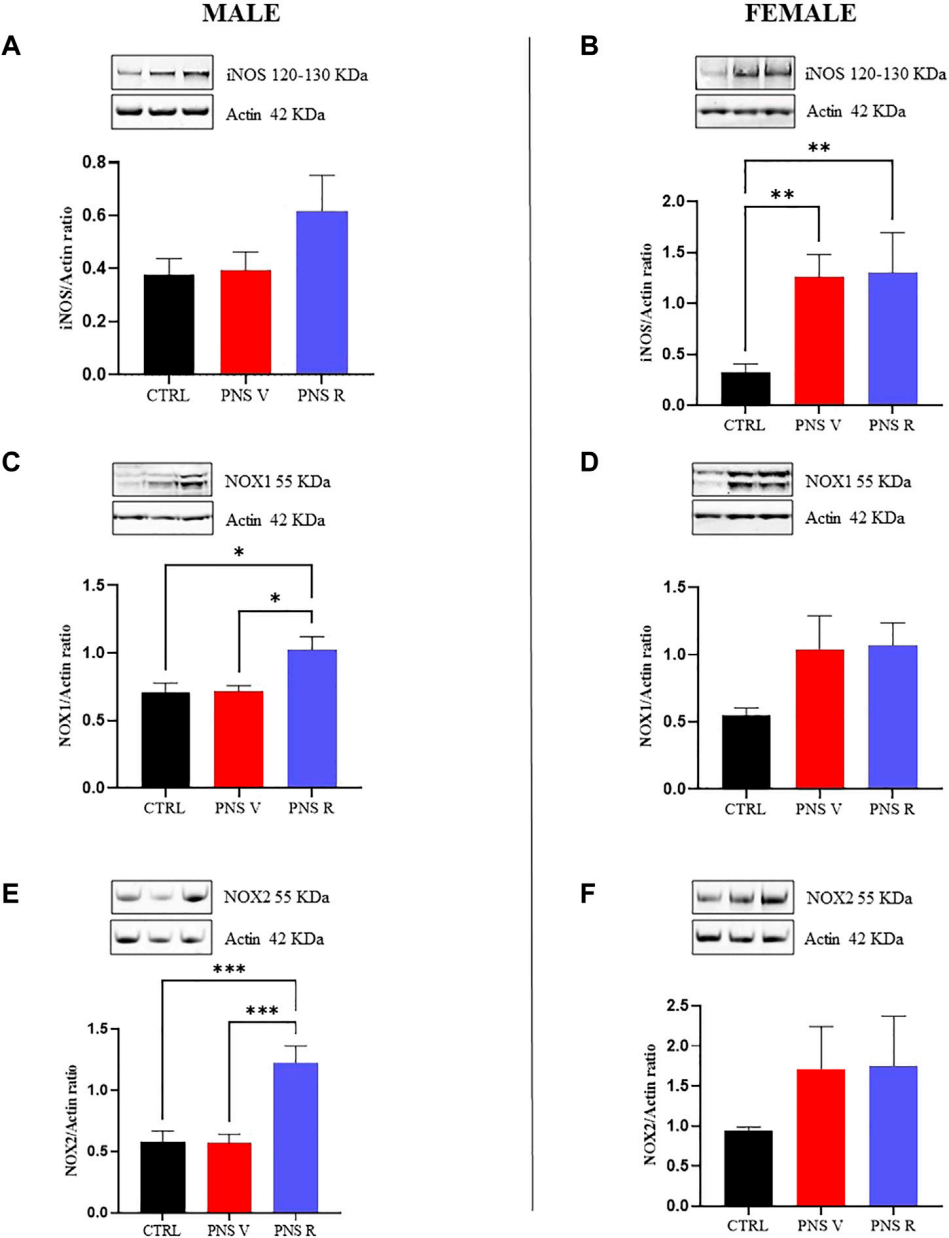
Oxidative stress response in vulnerable and resilient PNS rats. Effects induced by PNS exposure on iNOS, NOX1, and NOX2 expression in total protein extracts from hippocampus of CTRL and PNS rats evaluated by Western Blot analysis. Representative immunoblots and histograms of iNOS (120 kDa–130 kDa) in CTRL and PNS male **(A)** and female **(B)** adolescent rats (**p < 0.01 vs. CTRL). Representative immunoblots and histograms of NOX1 (55 kDa) in CTRL and PNS male **(C)** (*p < 0.05 vs. CTRL and PNS V) and female **(D)** adolescent rats. Representative immunoblots and histograms of NOX2 (55 kDa) in CTRL and PNS male **(E)** (***p < 0.001 vs. CTRL and PNS V) and female **(F)** adolescent rats. Densitometric values were normalized against actin used as internal control. All data are shown as mean ± SEM of CTRL male *n* = 10; PNS V male *n* = 9; PNS R male *n* = 5; CTRL female *n* = 10; PNS V female *n* = 5; PNS R female *n* = 4.

When we analyzed the oxidative response after PNS procedure and potential changes of pro-oxidant factors in hippocampus of PNS rats, we found an increased expression of NOX1 (Figure 5C, *p* < 0.05 vs. PNS V and CTRL), and NOX2 (Figure 5E, *p* < 0.001 vs. PNS V and CTRL) in male PNS R rats while a trend toward increased levels of iNOS protein was observed in the same group compared to both PNS V and controls (Figure 5A). The oxidative stress response to PNS exposure was significantly different in female rats, where we detected high and significantly increased levels of iNOS in both PNS V and PNS R (Figure 5B, *p* < 0.01 vs. CTRL), whereas both NOX1 (Figure 5D) and NOX2 (Figure 5F) showed a non-significant increase.

## 4 Discussion

Epidemiological and preclinical studies demonstrated that the exposure to stress or adverse life events during pregnancy increases the risk to neuropsychiatric disorders such as depression in the offspring. Indeed, PNS, especially during pregnancy, can lead to long-term effects in the offspring impacting on emotional, behavioral, and cognitive outcomes and increasing the risk of depression development in early adulthood (Sohr-Preston and Scaramella 2006; Plant et al., 2015; Brooker et al., 2020).

In this study, by employing a validated PNS animal model, we have found for the first time that the exposure to early adverse life events is able to induce a depressive-like phenotype paralleled by memory deficits more evident in adolescent female compared to male rats (Figure 1) and we also examined the percentage of vulnerable and resilient rats detected with the z-normalization, observing that male vulnerable rats are 62% compared to 68% of female vulnerable adolescent rats (Figure 2). Our data are in accordance with other studies reported in literature, where a positive association has been found among female offspring between prenatal maternal stress and cognitive dysfunction during early childhood (Simcock et al., 2017; Sutherland and Brunwasser 2018). Preclinical and clinical data also show that females are more susceptible to several types of stressful events leading to a higher prevalence of depression compared to male subjects (Schmidt et al., 2018; Kalinichenko et al., 2019).

Moreover, by employing the PNS animal model, previous studies have found memory deficits measured by NOR test in male and female rats at adulthood (PND73 and PND80, respectively) (Cattaneo et al., 2019), and the stressful events during the last week of gestation are able to induce in the offspring cognitive deficit, which in females is dependent on the estrous cycle phase (Moura et al., 2020). In our cohort of PNS adolescent male and female rats (PND 35) no differences were observed with regard to sucrose preference among all experimental groups. As recently highlighted by Markov (Markov 2022), this test could give inconsistent results which may be influenced by several factors such as differences in sucrose preference concentration threshold, water and food deprivation, and differences in animals’ susceptibility to stress. We cannot exclude that PNS exposure during pregnancy can impair the responsiveness in our cohort only in adult rats. Future studies are planned to evaluate the onset of anhedonic-like behavior in adult rats.

To understand the molecular mechanisms underlying the vulnerability or resilience to PNS, we focused on neuroinflammatory phenomena in the hippocampus of male and female adolescent rats exposed to PNS because previous studies have shown that early life stress exposure may trigger pro-inflammatory system activation in the brain, an event known to contribute to the development of depression (Ślusarczyk et al., 2016; Caruso et al., 2020). Several studies carried out in animal models of depression have shown increased levels of pro-inflammatory cytokines paralleled by decreased TGF-β1 levels in hippocampus (Torrisi et al., 2019). Furthermore, TGF-β1 signaling in the CNS plays a key role in mediating cellular and behavioral plasticity related to depression and the rescue of TGF-β1 canonical pathway in the hippocampus mediates behavioral effects of antidepressant treatment (Gergues et al., 2021; Mitra et al., 2022). In particular, a selective atrophy of the hippocampus, a brain area essential in the storage/consolidation of short-term memory and learning, has been found in depressed patients compared with healthy controls (Koolschijn et al., 2009), and, similarly, adolescent subjects at a higher risk to develop depression after early life adversity possess smaller hippocampal volumes (Rao et al., 2010). We found for the first time a gender-dependent deficit of hippocampal TGF-β1 levels which seems to contribute significantly to increase the vulnerability to depression, whereas the increased expression of TGF-β1 protein was able to promote resiliency in adolescent male rats (Figure 4). Our data are in agreement with a study carried out by Trojan et al., where a deficit of TGF-β1 gene expression was found in hippocampus and frontal cortex of adult rats previously exposed to PNS during pregnancy, paralleled by anxiety and a depressive-like phenotype, which were normalized by a chronic antidepressant treatment (Trojan et al., 2017).

When considering the differences in gender specific PNS effects on TGF-β1 pathway, but not on oxidative stress, we believe that it may depend on the bidirectional regulation existing between sex hormones and TGF-β1 and its receptor. In fact, it has been already demonstrated that TGF-β1 is involved in gonads and secondary sex organs development, spermatogenesis, and ovarian function (Ingman and Robertson 2002). Interactions exist between follicle-stimulating hormone (FSH), estradiol-17 beta, and TGF-β1 expression and function (Dorrington et al., 1993). Additionally, TGF-β1 secreted by astrocytes can regulate GnRH release *via* the SMAD-dependent pathway (Galbiati et al., 2005). In this context, it has been recently reported, by using an *in vivo* model of diabetic nephropathy, that male and female sex hormones seem to influence the TGF-β1/TGF-βR axis by different mechanisms; in particular estradiol *via* TGFβ-R1 and dihydrotestosterone *via* TGFβ-R2 (Ziller et al., 2020). Further studies are needed in the PNS model to understand the impact of estradiol-17 beta on TGF-β1 signaling in the brain.

The novelty of the present study stems from the evidence that we identified for the first time the deficit of TGF-β1 pathway as an early event in PNS-induced depression that can be detected in adolescent depression and might then represent a novel pharmacological target to reduce vulnerability to adult depression. Along this line, it has been recently demonstrated that children at 6 years of age with a lower gene expression score of TGFβ-R2 showed larger amygdala volumes in relation to greater prenatal maternal depressive symptoms (Qiu et al., 2021), suggesting a great impact of prenatal stress and prenatal depression on TGF-β1 pathway as a vulnerability factor for child and adolescent depression. In the present work we have focused our attention only on TGF-β1, starting from the evidence that a selective deficit of this cytokine/neurotrophic factor has been found in major depressed patients (Myint et al., 2005), whereas no studies both in humans and animal models of depression show a deficit of TGF-β2. We cannot exclude a role for TGF-β2 in depression, because this neurotrophic factor is expressed in the dentate gyrus and it is also known to modulate serotonin synthesis and metabolism (Chleilat et al., 2019).

In the present study, we planned to increase the translational value of our model by combining the analysis of TGF-β1 pathway in the hippocampus with ELISA assays on plasma samples in the same PNS rats. We found an increase in gene and protein expression levels of TGFβ-R2 in the hippocampus of both male and female resilient rats (Figures 3, 4), whereas at peripheral level we detected an increase of TGF-β1 only in vulnerable and resilient adolescent male rats (Figures 4E,F). Unfortunately, we did not find an increase of plasma TGF-β1 levels in female PNS R rats, suggesting that further studies are needed to understand whether plasma TGF-β1 levels can contribute or not to increase resiliency in female PNS rats.

It is known that the interactions between chronic inflammation and oxidative stress have been implicated in the pathophysiology of depression (Chauhan and Chauhan 2006; Berk et al., 2011; Capuron and Miller 2011). Neuroinflammation can trigger oxidative stress through several mechanisms, such as through the overproduction of free oxygen radicals by activated microglia and astrocytes, and oxidative stress can stimulate several transcription factors to induce a higher release of circulating cytokines (Closa and Folch-Puy 2004; Hayley et al., 2005). Moreover, starting from the evidence of an interaction between early life adverse experience and redox state dysfunctions in PNS-induced mental disorders such as depression (Schiavone et al., 2013), we focused our attention on the potential modulation of pro-oxidant factors in the hippocampus of PNS adolescent rats. In the present study, the modulation of iNOS, NOX1, and NOX2 pro-enzymes, under our experimental conditions, was measured at both gene and protein expression levels since they represent well-demonstrated markers of oxidative stress. In particular, as previously mentioned, iNOS is responsible for nitric oxide production, while NOX1 and NOX2 are responsible for superoxide production. It is also well-known that superoxide anions easily react with NO producing peroxynitrite, an extremely reactive and toxic molecule able to damage the four major classes of biological macromolecules (DNA, carbohydrates, fatty acids and proteins) and mitochondria (Caruso et al., 2021a). We have also recently demonstrated that oxidative stress, taking place as a consequence of pro-oxidant enzymes (i.e., iNOS and NOX2) activation (Caruso et al., 2021b), along with the previously showed deficit of TGF-β1 (Torrisi et al., 2019), is responsible for depressive-like phenotype in an animal model of amyloid-induced depression. We also recently showed as the rescue the TGF-β1 pathway can contribute to prevent amyloid-induced depression and cognitive decline by counteracting oxidative stress. Interestingly, we found that the oxidative stress response to PNS exposure was significantly different in female compared to male rats (Figures 3, 5). Several studies reported that the exposure to early life stress conditions (e.g., maternal deprivation) can influence the brain functions in the offspring and can lead to an increase in oxidative stress at the CNS level (Mumtaz et al., 2018; Safarpour et al., 2021). Furthermore, it has been demonstrated that rats exposed to social isolation at weaning (PND 21) show an increase in NOX2 expression associated with high levels of oxidative stress markers (Schiavone et al., 2009). In this context, we found a strong increase in the expression of iNOS in both PNS V and PNS R female rats compared to control animals (Figures 3F, 5B), (Figures 3E, 5A). In addition, NOX2 showed a significant increase in the hippocampus of male PNS R rats compared to PNS V rats at gene and protein level (Figures 3I, 5E); conversely, NOX1 was significant increased only at protein level in male PNS R rats (Figures 5D,F). These data suggest a prominent activation in the oxidant response following stress exposure in early life and that initial differences in pro-oxidant markers levels may determine the individual characteristic response to chronic stress. In this context, it has been demonstrated that NOX2 gene is under the control of TGF-β1 pathway and activated SMAD signaling (Zhang et al., 2014) and then we can hypothesize that an increased activation of TGF-β1 pathway in male resilient rats can promote the increased expression of NOX2 in PNS R male rats. Further studies in neuronal and glial cell cultures as well as in animal models of depression are needed to validate this hypothesis.

Our study shows some limitations, because it is a correlative study rather than a mechanistic one, and future studies are needed to understand whether TGFβ-R2 silencing or lowering TGF-β1 level can increase susceptibility of rats to PNS-induced depressive like behavior. Further studies are also needed, in the PNS model, to understand the impact of gender and estradiol-17 beta on TGF-β1 signaling in the brain. Our study has analyzed the impact of PNS procedure on adolescent rats, but we cannot exclude possible long-term consequences of exposure to PNS into adulthood. Finally, we did not find a correlation between the central and the peripheral levels of TGF-β1 in both male and female PNS rats. It is also possible that no correlation exists between a central deficit of TGF-β1 after PNS procedure and the reduced levels of this cytokine in the periphery. A recent study conducted in non-human primates, exposed to two consecutive acute confinement stress periods, seems to suggest this hypothesis, with the stress condition able to decrease TGF-β1 concentrations only in cerebrospinal fluid, but not in serum (Coplan et al., 2017).

Overall, our data, obtained in animal model of PNS, indicate that PNS procedure during the last week of gestation can induce in female adolescent rats a depressive-like phenotype combined with recognition memory deficit mimicking both affective and cognitive symptoms observable in adolescent depression. We demonstrated for the first time that an impairment of the TGF-β1 pathway as well as oxidative stress can contribute to increase the vulnerability to adolescent depression induced by PNS and suggest that the rescue of TGF-β1 signaling might represent a new pharmacological strategy to increase resiliency to chronic stress.

## Data Availability

The original contributions presented in the study are included in the article/Supplementary Material, further inquiries can be directed to the corresponding author.
